# Macrophages are necessary for skin regeneration during tissue expansion

**DOI:** 10.1186/s12967-019-1780-z

**Published:** 2019-01-21

**Authors:** Jianke Ding, Lei Lei, Shiqiang Liu, Yu Zhang, Zhou Yu, Yingjun Su, Xianjie Ma

**Affiliations:** Department of Plastic and Reconstructive Surgery, Xijing Hospital, Fourth Military Medical University, 127 Chanle West Road, Xi’an, 710032 Shaanxi Province China

**Keywords:** Macrophages, Skin regeneration, Mechanical stretch, Tissue expansion

## Abstract

**Background:**

Tissue expansion is a procedure that promotes skin regeneration by mechanical stretch. During the stress and relaxation cycle, the skin undergoes a repeated microtrauma which triggers an immune response leading to the recruitment of macrophages to repair the damaged tissue. Macrophages have been found to be necessary for tissue repair and wound healing, but their effects on skin regeneration during mechanical stretch remain unclear.

**Methods:**

The dynamic changes of macrophages in the rat skin tissues undergoing expansion were quantitatively determined by immunohistochemistry staining. The area of the expanded skin, skin thickness, dermal collagen density, cell proliferation and tissue vascularization were examined to determine the effects of macrophages on the expanding skin. The phenotypes of macrophages and the growth factors related to skin regeneration were also examined to evaluate the underlying mechanisms for the involvement of macrophages in skin regeneration. As a comparison, the tissue samples of expanding skin in which the macrophages were depleted by topically utilizing clodronate liposomes were also evaluated.

**Results:**

The number of skin macrophages in skin maintained in the high level during the skin expansion compared to non-expanded skin. We found that a switch from an M1- to M2-dominant response during tissue expansion. After the macrophages were depleted, the skin regeneration was inhibited, as evidenced by a smaller expansion area, thinner skin layers and decreased cell proliferation rate, collagen synthesis and, skin vascularization. The secretion of epidermal growth factor (EGF), fibroblast growth factor (FGF), and vascular endothelial growth factor (VEGF) were decreased when macrophages were depleted.

**Conclusions:**

Our findings suggest that macrophages are necessary for skin regeneration during tissue expansion. Modulating inflammation may provide a key therapeutic strategy to promote skin growth under mechanical strain.

**Electronic supplementary material:**

The online version of this article (10.1186/s12967-019-1780-z) contains supplementary material, which is available to authorized users.

## Background

Reconstruction of large skin defects resulting from different causes remains one of the biggest challenges in reconstructive surgery. Tissue expansion is a procedure to harvest large skin tissue in response to mechanical force by implanting a silicone expander subcutaneously and continuous injecting saline into it. This process, first described by Neumann [1] and later improved by Radovan [2] and Austad et al. [3], is one of the most important surgical techniques in plastic and reconstructive surgery. Skin tissue expansion can harvest enough “extra skin tissue” which is similar in texture, color and flexibility to the lost tissue to reconstruct large skin defects after trauma, tumor excision or infection. However, multiple surgical procedures, low expansion efficiency, and high complication rates limit its application [4, 5]. Therefore, the mechanism by which skin responds to mechanical strain remains to be elucidated.

During the expansion period, the skin undergoes stress and relaxation as well as injury and repair. Immune cells such as macrophages rush to the wounded site to clear debris and repair the damage [6]. Unlike wound healing which results in scar tissues, skin tissue expansion could regenerate new skin rather than simply stretch the pre-expansion skin [7]. A better understanding of how skin regenerates under such microenvironment will allow the improvement of current surgical techniques. However, the immune system specifically the macrophages responses in the skin tissue under mechanical stretch is not well characterized during this process.

Recent researches have been shown that macrophages play a key role to initiate tissue regeneration in highly regenerative animals such as zebrafish [8] and salamander [9]. Unlike these animals, few mammals can renew a damaged tissue or regenerate extra tissues. However, tissue expansion is a special model to promote skin regeneration without scar formation in mammals including human being. Until now, it was not clear how the mechanical strain promotes the skin regeneration.

To better understand the role of macrophages and how new tissue regenerates in tissue expansion model, we compared the number of macrophages between expanded and non-expanded skin and examined the role of macrophages by utilizing a rat model wherein the tissue macrophages were kept intact or depleted. Our results clearly demonstrated that macrophage depletion inhibits skin regeneration during tissue expansion determined by quantitation of skin area, skin thickness, collagen synthesis, cell proliferation, and vascularization. The underlying mechanisms associated with skin regeneration were also explored. Our findings suggest that therapeutic modulation of macrophages might facilitate regeneration of expanded skin.

## Methods

### Tissue expansion model

Adult male Sprague–Dawley rats (Experimental Animal Center of the Fourth Military Medical University, China) weighing 120 to 150 g were randomly divided into 4 groups: group 1 (n = 16), expanded group without treatment; group 2 (n = 17), non-expanded group without treatment served as a control; group 3 (n = 18), expanded group with clodronate liposomes- or PBS liposomes treatment; group 4 (n = 17), non-expanded group with liposomes treatment served as a sham control. The animal experiments were approved by Experimental Animal Committee of the Fourth Military Medical University. All groups were implanted subcutaneously with tissue expanders on day 0. The tissue expansion model was previously described [10]. Briefly, silicone expanders (10 ml, Weining, Shanghai, China) were implanted subcutaneously into the backs of rats. The border of two symmetrical 1.0 × 1.5 cm rectangle areas on bilateral of the expanded flap were tattooed for tracing the expanding areas. For group 1 and group 3, routine inflation was carried on every 2 days from the post-operation day 7, until the intracapsular pressure reaches 60 mmHg. Group 2 and group 4 were served as sham control groups without inflation.

### Clodronate liposome treatment

According to the previously reported protocols [11–13], 40 ul of 50 ug/ml PBS liposomes or clodronate liposomes (http://www.clodronate-liposomes.org) were subcutaneously injected into the left or right side of the tattooed skin area on post-operation days 0, 3, and 10. Tissue biopsies were collected at days 7, 14, and 35, respectively, for pathologic examination in both groups 3 and 4.

### Histological evaluation

Tissue specimens were collected, fixed in 4% paraformaldehyde for 24 h and embedded in paraffin. Tissue sections of 4 μm were cut and stained with hematoxylin and eosin (H&E) and Masson trichrome according to routine procedures. The thickness of skin was measured on H&E stained slides. As for immunohistochemistry, sections were submitted to an antigen retrieval step at 96 °C for 20 min in citrate buffer at PH 6.0, blocked for 1 h and primary antibody was applied overnight at 4 °C in a humid chamber: mouse anti-rat CD68 (Abcam, Cambridge, UK), rabbit anti-rat inducible nitric oxide synthase (iNOS, Abcam, Cambridge, UK), rabbit anti-rat CD206 (Abcam, Cambridge, UK), mouse anti-rat PCNA (Abcam, Cambridge, UK) and isolectin GS-IB4 (Invitrogen, Carlsbad, CA, USA). Fluorescently conjugated secondary antibodies (antibody to mouse or rabbit) were applied for 3 h at room temperature in a humid chamber (1:400, Invitrogen, Carlsbad, CA, USA). Nuclei were counterstained with 10 ug/ml DAPI (Invitrogen, Carlsbad, CA, USA).

### Skin regeneration efficiency evaluation

The tattooed area of either side of each expanded skin area was measured on days 14, 28, and 35, respectively, and the relative skin thicknesses of the skin biopsy samples (dermis plus epidermis) collected at days 14 and 35 were calculated by using ImageJ. The Masson staining positive areas were analyzed by Image-Pro Plus 6.0, and the average percentage of the stained area was calculated. All data were collected by a blinded observer.

### Transcutaneous oxygen pressure evaluation

Since oxygen is carried by the blood, transcutaneous oxygen pressure (TcPO_2_) can be used as an indirect measure of blood flow to expanded skin tissue. TcPO_2_ of the expanded skin flap were monitored at days 14 and 35, respectively, following the manufacturer’s instruction. Briefly, following two calibrations, an electrode that was pre-heated to 43 °C was placed on the center of the tattooed skin after being shaved and cleaned with alcohol. A stabilization period of 20 min with TcPO_2_ changes less than 2 mmHg in 5 min was allowed before recording basal values.

### Polymerase chain reaction analysis

Total RNA was extracted from expanded skin treated with PBS liposomes or clodronate liposomes and normal skin using Trizol reagent (Invitrogen, Camarillo, CA, USA). Briefly, 80–100 mg of skin tissues were homogenized in 1 ml of Trizol reagent per 100 mg of tissue. Post-centrifugation, RNA was extracted with chloroform and precipitated with isopropyl alcohol. Isolated RNA samples were then reverse transcribed into cDNA using a cDNA synthesis kit (Takara, Shiga, Japan) following standard protocols. Quantitative gene expression was performed using synthetic primers and SYBR Green (Takara, Shiga, Japan). The sequences were as follows: Rat vascular endothelial growth factor (VEGF), 5′-GCACGTTGGCTCACTTCCAG-3′ (sense) and 5′-TGGTCGGAACCAGAATCTTTATCTC-3′ (antisense); rat epidermal growth factor (EGF), 5′-TTCCAAACGCCGCAGACTTAC-3′ (sense) and 5′-TGGGATAG CCCAATCCGAGA-3′ (antisense); rat basic fibroblast growth factor (bFGF), 5′-GAGGAGTTGTGTCCATCAAGG-3′ (sense) and 5′-CGTTTCAGT GCCACATACCA-3′ (antisense); Rat transforming growth factor, beta (TGF-β),5′-CATTGCTGTCCCGTGCAGA-3′ (sense) and 5′-AGGTAACGCCA GGAATTGTTGCTA-3′ (antisense); rat actin, beta (β-actin); 5′-GGAGATTACTGCC CTGGCTCCTA-3′ (sense) and 5′-GACTCATCGTACTCCTGCTTGCTG-3′ (antisense). Relative mRNA levels were normalized to β-actin and expressed as a relative unit.

### Statistical analysis

Statistical analyses of differences between groups were performed by a Student t-test or two-way ANOVA. Data are always shown as mean values ± SD. P values less than 0.05 were considered statistically significant. In all cases, the varied levels of statistical significances were labeled as *p ≤ 0.05; **p ≤ 0.01; ***p ≤ 0.001, and ****p ≤ 0.0001.

## Results

### Dynamic changes of tissue macrophage density during the tissue expansion

**Fig. 1:**
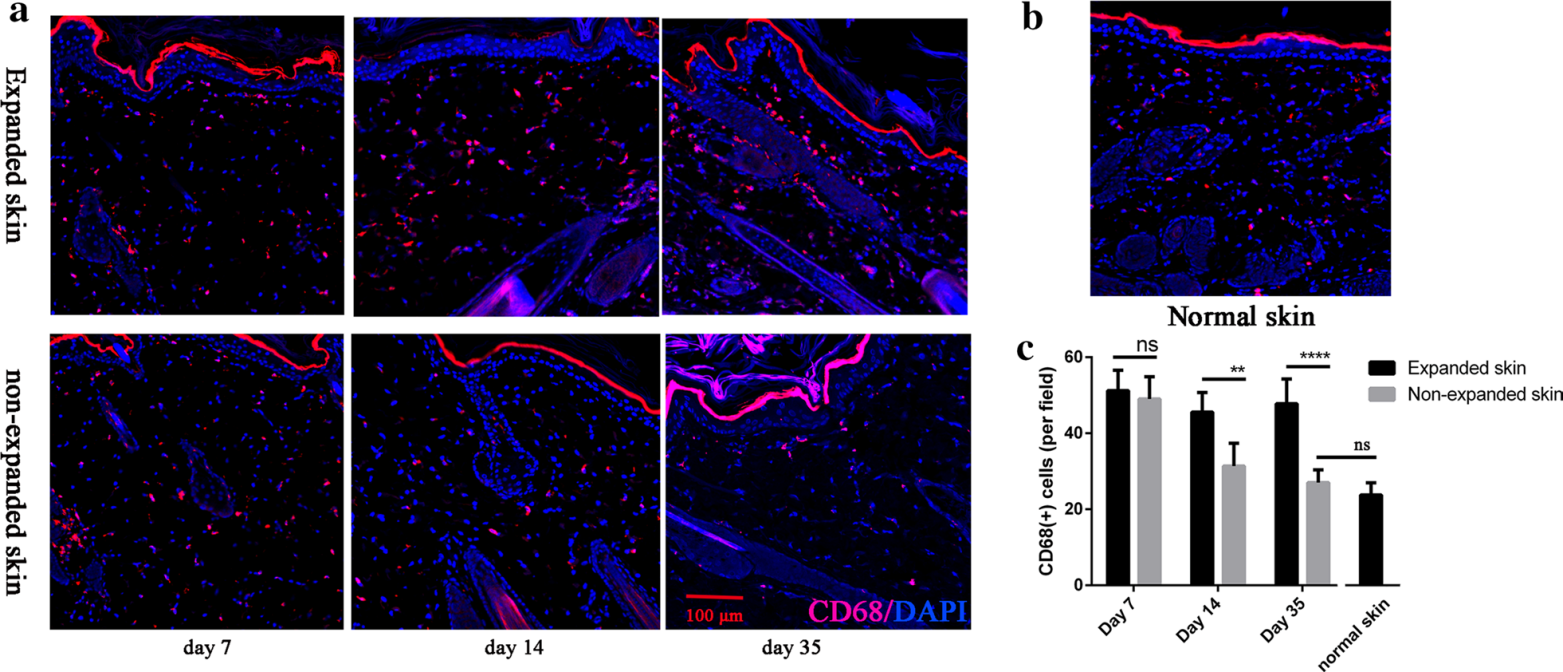
The number of skin macrophages maintained in the high level during a mechanical stretch. **a** Skin sections were stained with anti-CD68 (red) to visualize macrophages in expanded and non-expanded skin at day 7, day 14 and day 35. **b** Normal skin sections stained with anti-CD68. **c** The total number of macrophages in the skin was quantified on 3 sections of 5–6 rats per time point. The expanded skin had more total macrophages than the non-expanded skin. Data represent mean ± SD. **p ≤ 0.01; and ****p ≤ 0.0001

To evaluate the differences between the expanded and non-expanded skin macrophage response, we sought to count the number of macrophages in the skin using immunostaining. Skin sections from tissue samples collected at day 7 through day 35 during the expansion period were stained for markers for macrophages (CD68) (Fig. 1a). There was no significant difference in the number of macrophages between the expanded and non-expanded group on day 7 when the expansion processes began. However, the densities of macrophages were significantly higher in the expanded skin tissues on days 14 (p < 0.01) and 35 (p < 0.01, Fig. 1c), respectively, relative to that of non-expanded tissues. And there was no difference in the number of macrophages between non-expanded skin at day 35 and the normal skin (p > 0.05, Fig. 1b, c). Therefore, mechanical strain maintained more macrophages in the skin, suggesting macrophages play a role during tissue expansion.

### Depleting macrophages in the tissue expansion model

**Fig. 2:**
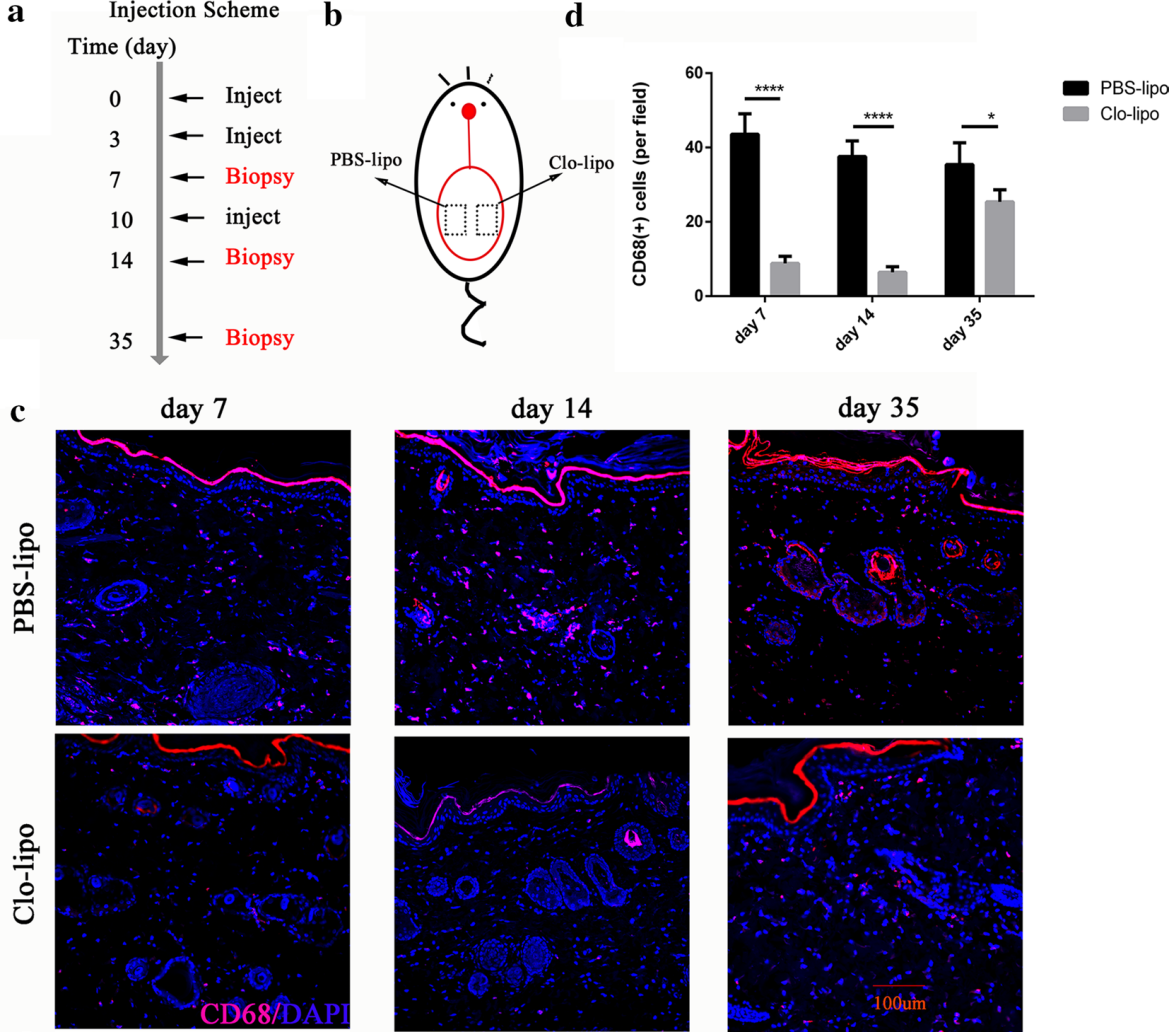
Macrophages depletion in the tissue expansion model following clodronate liposomes injection regimen. **a** Skins were injected with clodronate liposomes (Clo-lipo) or PBS liposomes control (PBS-lipo) at day 0 immediately after expander implantation, day 3 and day 10. Skin tissues were collected at day 7, day 14 and day 35. **b** Clo-lipo or PBS-lipo were subcutaneously injected on the right or left side of the tattooed expanded skin (1.5 cm × 1 cm) at indicated days. **c** Skin sections were stained with anti-CD68 (red) to visualize macrophages in Clo-lipo and PBS-lipo treated expanded skin at day 7, day 14 and day 35. **d** Morphometric quantification of macrophages in the expanded skin at indicated time points following expander implantation (at least 5 animals at each time point). *p ≤ 0.05, ****p ≤ 0.0001

In order to investigate the role of macrophages during skin tissue expansion, we used a topical macrophages depletion model by using clodronate liposomes. As shown in Fig. 2b, two symmetrical 1.0 × 1.5 cm squares located on bilateral sides of the expanded flap were tattooed on their borders. On one side, the clodronate liposomes were injected subcutaneously at days 0, 3, and 10, respectively, to deplete skin tissue macrophages. The other side, which served as the control, received an injection of PBS liposomes of the same dosage. Immunofluorescence staining of the tissue sections with the treatment of clodronate liposomes showed a marked reduction in the macrophage population at days 3 (p < 0.0001), 14 (p < 0.0001), and 35 (p < 0.05), respectively.

### Macrophage depletion hinders skin regeneration during tissue expansion

**Fig. 3:**
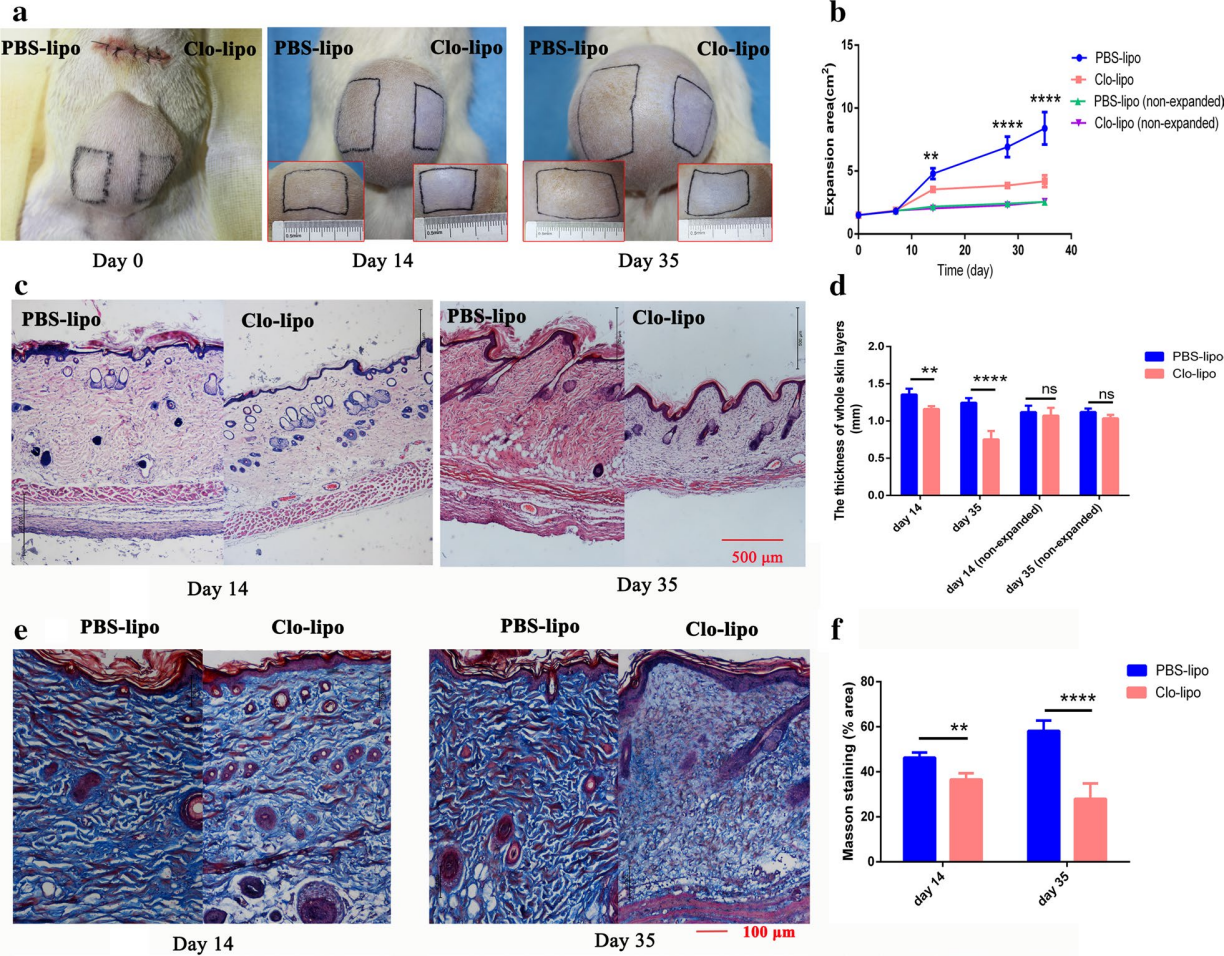
Macrophage depletion with clodronate liposomes inhibits skin regeneration during tissue expansion. **a** Representative expansion area from PBS-lipo (left) and Clo-lipo (right) treated skin followed over time. **b** Expansion area of the two sides was measured over time. The difference appeared at day 14 (n = 13) and became more evident at day 28 and day 35 (n = 8). **c**, **d** H&E staining showed a thicker whole skin thickness in PBS-lipo side than that in Clo-lipo side at days 14 (n = 5, p < 0.01) and day 35 (n = 8, p < 0.0001). **e**, **f** Masson’s trichrome stained Clo-lipo expanded skin at day 14 (n = 5, p < 0.01) and day 35 (n = 8, p < 0.0001) showed a remarkable reduction in collagen density than that of PBS-lipo expanded skin. PBS-lipo, PBS liposomes; Clo-lipo, clodronate liposomes. **p ≤ 0.01; and ****p ≤ 0.0001

To evaluate the effects of macrophages on the expanded skin tissue, the expansion area, complications, and the thickness of skin tissue were measured. The skin tissues in the macrophages depletion side showed less expansion area than that of the control side. The difference was significant from 14 days (p < 0.01, n = 13) after surgery and became more evident on day 28 and day 35 (n = 8, p < 0.0001, Fig. 3a, b). Interestingly, histopathologic examination revealed that macrophages depleted skin in the expansion group displayed thinner skin thickness at days 14 and 35 respectively than that in control skin (Fig. 3c, d). One animal (1/8) suffered from expander exposure on macrophages depletion side during the expansion (Additional file 1). There was no significant difference in skin thickness or expansion area between macrophages depletion side and the control side in the non-expansion sham group (Fig. 3d).

Next, we determined the collagen density in the skin samples through microscopic evaluation of Masson’s trichrome stained tissue sections. The control expanded skin showed a significant increase in collagen density than that in the macrophages depleted skin at day 14 (p < 0.01) and day 35 (p < 0.0001, Fig. 3e, f), respectively, suggesting that macrophages play a role in collagen synthesis during tissue expansion.

### The effects of macrophages on cell proliferation and tissue vascularization during skin expansion

**Fig. 4:**
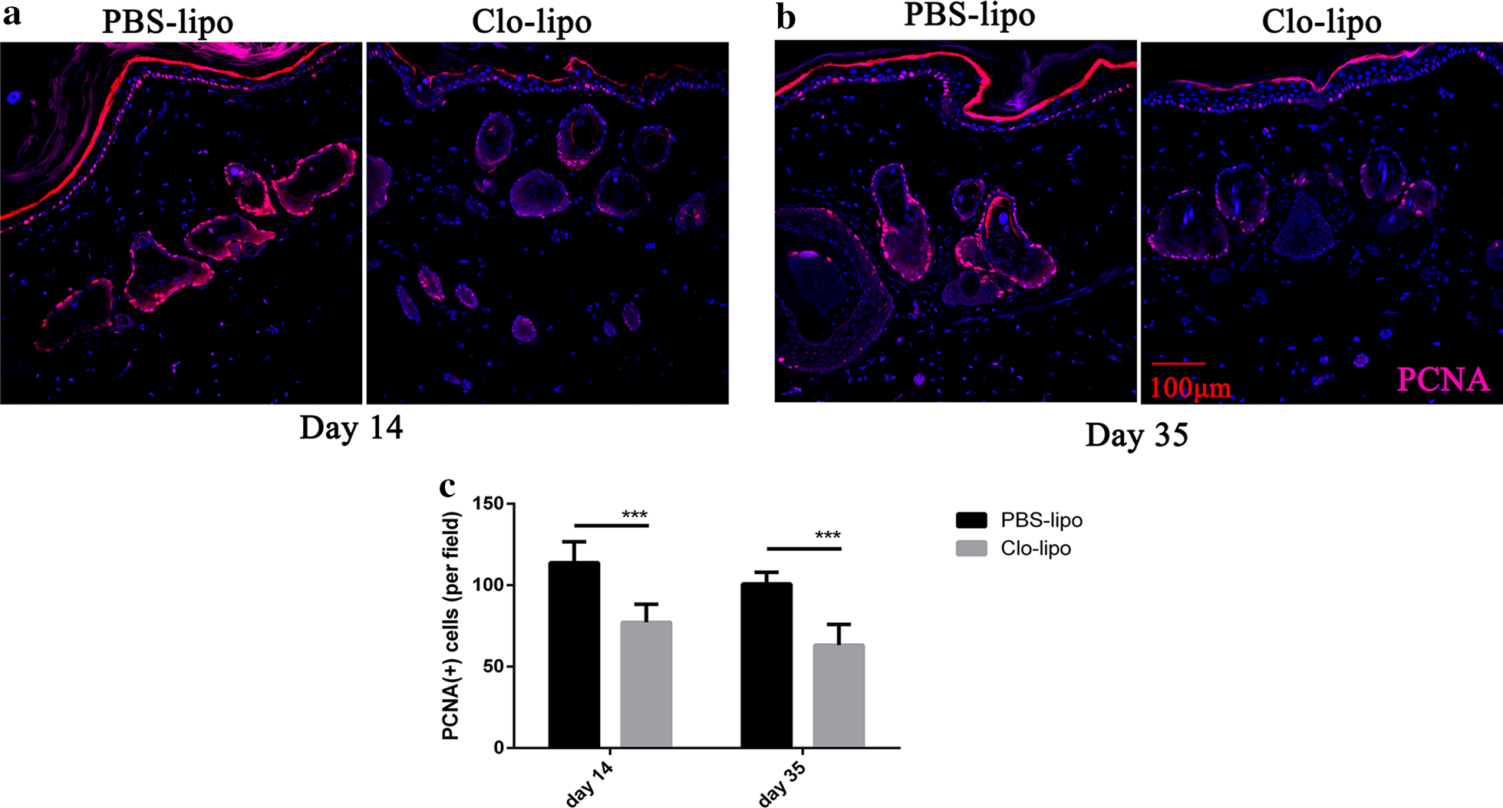
The effect of macrophage depletion on tissue cell proliferation during the process of tissue expansion. **a**, **b** Representative photographs of PCNA-positive cells (red) in the expanded skin. PCNA positive cells mainly located at the basal layer and hair follicles. **c** The total number of PCNA-positive cells in PBS-lipo side was higher than that in Clo-lipo side at day 14 (n = 5, p < 0.001) and day 35 (n = 8, p < 0.001). PCNA, anti-proliferating cell nuclear antigen; PBS-lipo, PBS liposomes; Clo-lipo, clodronate liposomes. ***p ≤ 0.001

To observe proliferating cells in the skin tissue samples, especially in the basal layer and around hair follicle where stem cells located, immunohistochemistry staining of anti-PCNA was employed. As shown in Fig. 4, PCNA-positive cells distributed all over the expanded skin but mainly concentrated in the basal layer and hair follicles. The control expanded skin revealed more PCNA-positive cells per field than that of the macrophages depleted skin at day 14 and day 35 (p < 0.001, Fig. 4a–c), respectively.

**Fig. 5:**
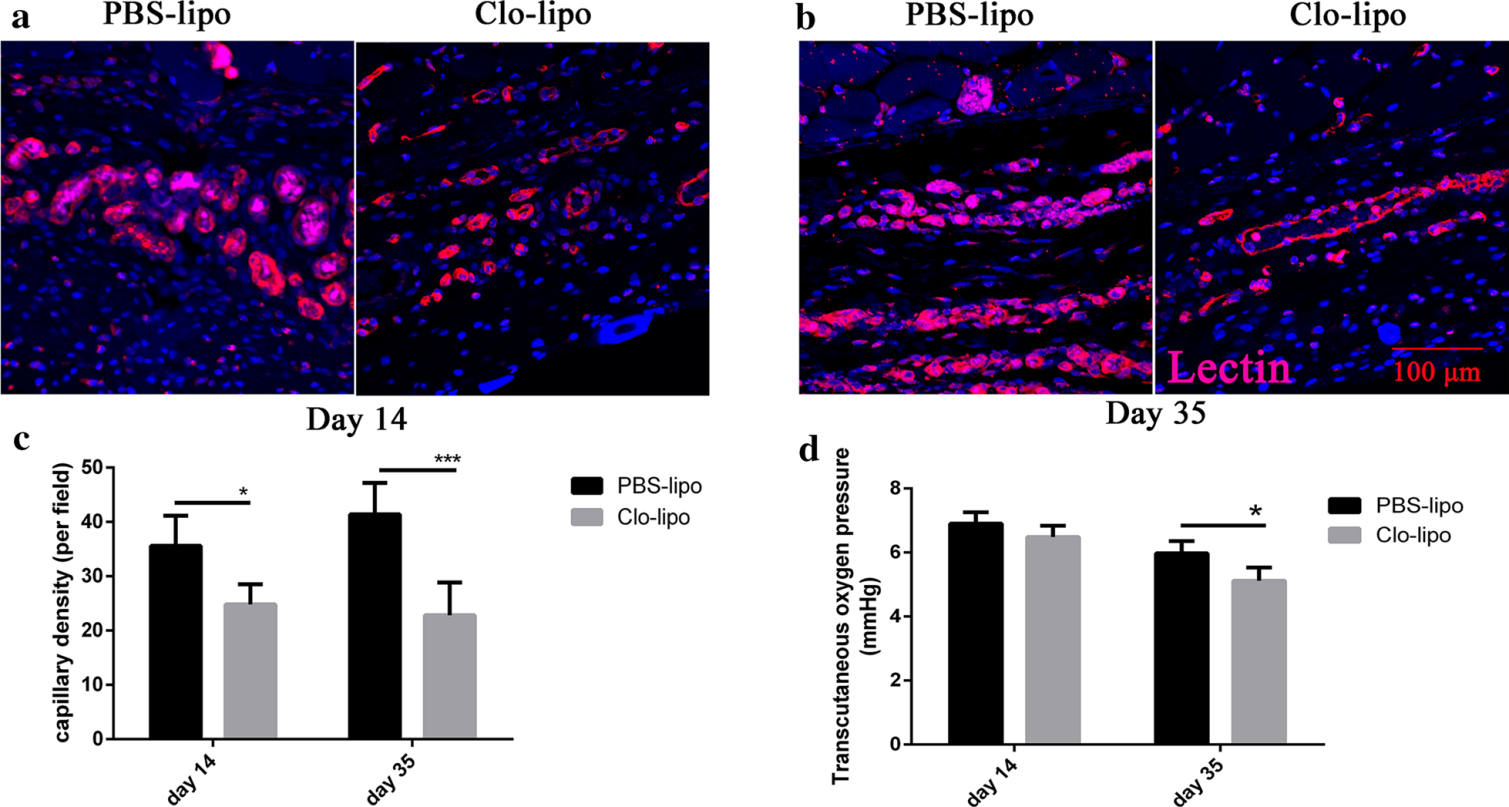
Angiogenesis is impaired in the macrophage-depleted expanded skin. **a**, **b** Sections from control or clodronate liposomes treated expanded skin stained with Lectin (red) and DAPI (blue) to label endothelial cells and nuclei, respectively, 14 days and 35 days after expander implantation. Vessels are visible in skin sections of the representative photographs. **c** Significantly fewer vessels are present in macrophage-depleted expanded skin compared with that in control skin at day 14 (n = 5, p < 0.05) and day 35 (n = 8, p < 0.001). **d** Transcutaneous oxygen pressure showed a higher oxygen pressure in control expanded skin at day 35 (n = 8, p < 0.05)

Isolectin immunohistochemical staining was employed to determine the degree of vascularization of the expanded flap tissues. The macrophages depleted skin had a lower degree of vascularization than that of the control expanded skin at day 14 and day 35 (p < 0.0001, Fig. 5), respectively. The measurements of the transcutaneous oxygen pressure showed a higher oxygen pressure in the control expanded skin at day 35 (p < 0.05, Fig. 5d).

### An M1-to-M2 switch during tissue expansion

**Fig. 6:**
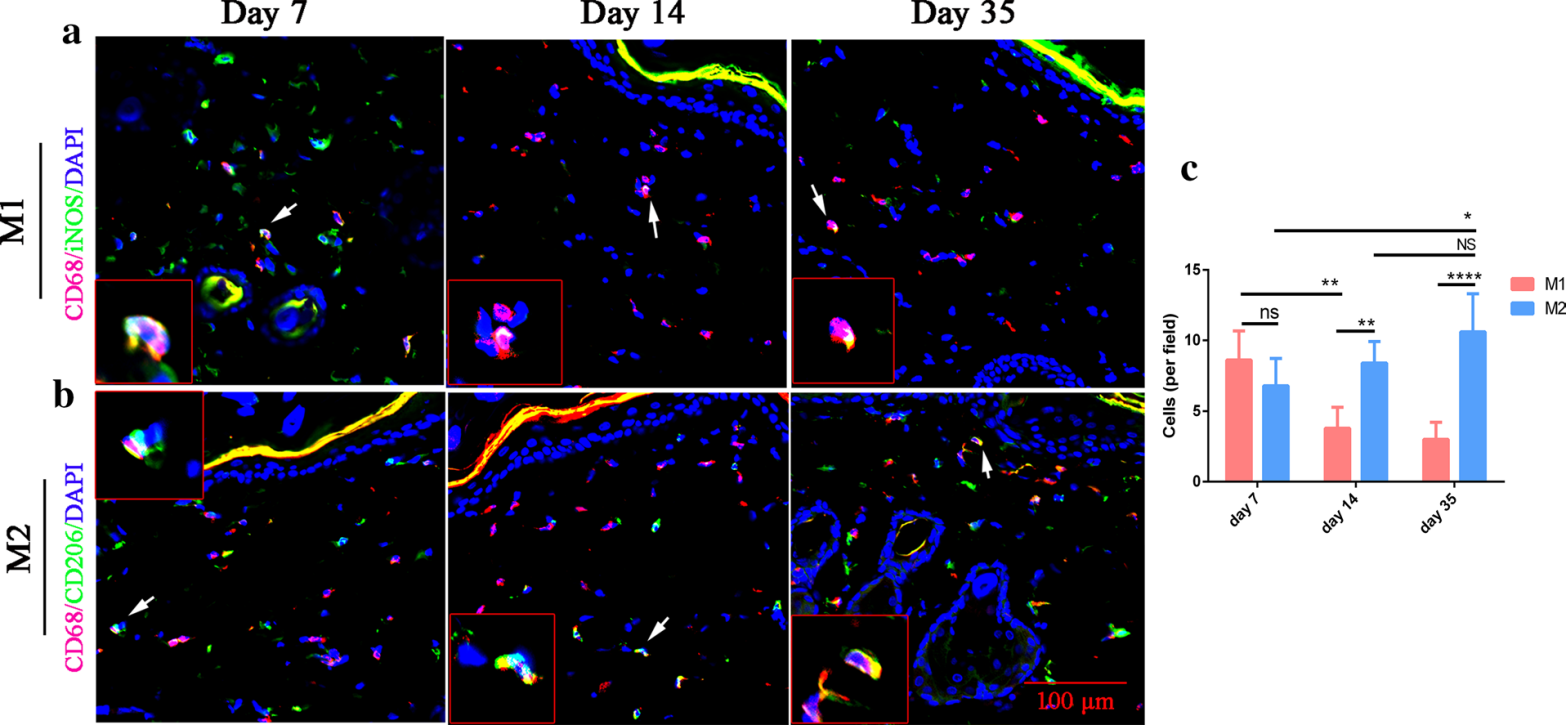
A switch from M1- to M2-dominant macrophage response occurs during tissue expansion. **a** Expanded skin stained for M1 marker iNOS (green) and pan-macrophage marker CD68 (red) at day 7, day 14 and day 35. **b** M2 marker CD206 (green) and pan-macrophage marker CD68 (red) were stained at day 7, day 14 and day 35. Arrows point to M1 or M2 macrophages. Insets show high-magnification images of the M1 or M2 cells. **c** The number of iNOS^+^ CD68^+^M1 cells and CD206^+^CD68^+^ per field in skin ± SD. iNOS, inducible nitric oxide synthase. Five rats sacrificed on day 7, 5 rats sacrificed on day 14 and 6 rats sacrificed on day 35 for statistical analysis. *p ≤ 0.05; **p ≤ 0.01; and ****p ≤ 0.0001

To understand how macrophages participate in skin regeneration, we sought to identify the phenotypes of macrophages during the tissue expansion. Tissue section staining with the traditional M1 marker (iNOS) or M2 marker (CD206) in combination with pan-macrophage marker CD68 was used to distinguish each macrophage phenotype in the skin tissues. At day 7 when the expansion process just began, there was no difference in the cellular number between M1 and M2 macrophage subtypes. However, by day 14 and day 35, a substantially higher density of CD68^+^/CD206^+^ double-positive M2 macrophages than that of the CD68^+^/iNOS^+^ double-positive M1 macrophages was revealed (Fig. 6c). The cellular density of M1 macrophages dropped significantly at day 14 (p < 0.01) and day 35 (p < 0.0001), respectively, compared to that at day 7. In contrast, the cellular density of M2 macrophages markedly increased during tissue expansion at day 35 (p < 0.05) relative to that at day 7.

### Macrophage depletion induced reduction of growth factor expression in expanded skin

**Fig. 7:**
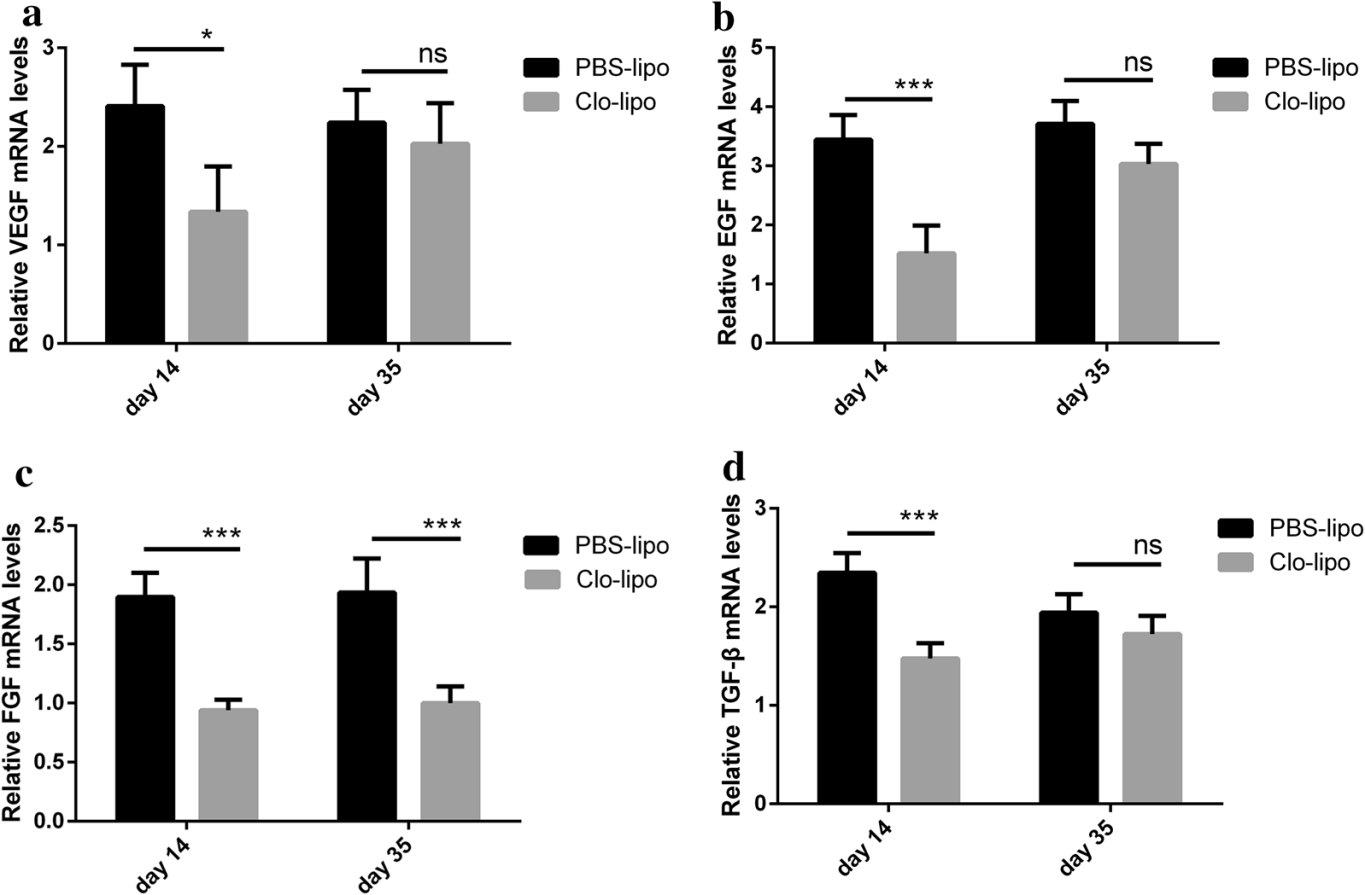
Macrophage depletion reduced growth factors in the expanded skin. Total RNA was extracted from the expanded skin with PBS liposomes or clodronate liposomes and normal skin, quantitative real-time PCR was employed to quantify the expression of VEGF, EGF, bFGF, and TGF-β. VEGF (**a**), EGF (**b**), bFGF (**c**) and TGF-β (**d**) dramatically decreased in macrophages depleted skin compare to control expanded skin at day 14. On day 35, only bFGF showed a decrease in macrophages depleted skin (**c**). Results are representative of three separate experiments from three animals in each group. VEGF, vascular endothelial growth factor; EGF, epidermal growth factor; bFGF, basic fibroblast growth factor; TGF-β, transforming growth factor, beta

Previous studies revealed that the growth factors such as VEGF, EGF, bFGF, and TGF-β are related to skin regeneration during tissue expansion [7, 14]. In our study, we used quantitative real-time PCR (qPCR) analysis to compare the expressions of these genes. As shown in Fig. 7, the expressions of VEGF, EGF, bFGF, and TGF-β dramatically decreased in the macrophages depleted skin compared to that of the control expanded skin at day 14. On day 35, however, only bFGF showed a decrease in the expression level in the macrophages depleted skin.

## Discussion

The ideal tissue for large defects reconstruction can be achieved through tissue expansion of the adjacent skin that has the similar texture and color to the lost tissue. It has been six decades since the tissue expansion technique was first described [1]. However, the precise mechanism of how the skin regenerates during the expansion remains ill-defined and the application of this technique is still limited for a few of donor sites such as neck and face [15]. Therefore, a better understanding of the cellular and molecular mechanism underlying the structural remodeling of the expanded skin may help therapeutic modulation to enhance skin regeneration.

Tissue expansion is pathologically similar to the repeated microtrauma processes that create a unique immunological microenvironment [16]. We found that the number of macrophages in the expanded skin tissues maintained in the high level during the skin expansion compared to that of the non-expanded skin. Numerous studies in the past showed that macrophages played a crucial role in tissue repair and wound healing [17–19]. More recently, in vivo depletion of macrophages using clodronate liposomes during adult salamander limb regeneration and African spiny mice ear regeneration supports the requirement for these cells to stimulate a regenerative response [9, 12]. Unlike these animals which have the extraordinary regenerative ability, few other mammals renew a damaged tissue. However, the mechanical stretch is commonly used in distraction osteogenesis and tissue expansion for regenerate extra tissues in clinical practice [20–22]. How the macrophages respond to mechanical stretch remains to be elucidated. In the present study, we showed the depletion of macrophages during tissue expansion, which provided a sustained mechanical stretch, inhibited skin regeneration. Our results suggest that this effect is associated with cell proliferation, collagen synthesis, flap tissue vascularization, and the change in growth factors expressions.

In response to the mechanical stretch, cells of epidermis and dermis receive the signals and begin to proliferate [23]. But how immune cells, especially macrophages, participate in this process remains unclear. Castellana et al. found that skin-resident macrophages contribute to the activation of skin epithelial stem cells and regulate their regenerative activity [11]. More recently, Wang et al. showed that macrophages within wound tissues activate hair follicle stem cells leading to the hair follicle regeneration [24]. Seven days following mechanical stretch, the macrophages appeared more abundant in the expanded skin than that of the non-expanded skin in our study. When these macrophages were depleted, less PCNA-positive cells were found in the macrophages depletion skin than that of the control skin, especially in the basal layer and in the follicle bugle of the epidermis. These results suggest that the macrophages in the expanded skin may contribute to the activation of the epidermal stem cells and enhance skin growth in our tissue expansion model.

The establishment of adequate blood flow is a key aspect to ensure tissue regeneration [25]. Previous studies have demonstrated the critical role of macrophages in angiogenesis of different models [26, 27]. To determine whether the macrophages mediate blood vessel formation during tissue expansion, we visualized the Isolectin stained vasculature in the tissue sections from either the control or clodronate liposomes treated skin. The results revealed more Isolectin-positive vessels in the control skin at days 14 and 35 compared with that in clodronate liposomes treated skin. Transcutaneous oxygen monitoring showed an increased oxygen pressure in the control skin than that in clodronate liposomes treated skin at day 35. Together, these data suggest macrophages contribute to skin tissue vascularization during the tissue expansion process.

Macrophages are phagocytic cells which maintain homeostasis and shape the immune response through the clearance of apoptotic cells and the production of soluble active factors [28]. Depending on the stages of polarization, macrophages can be subdivided into classically activated M1 or alternatively activated M2 phenotypes. M1 macrophages are known to secrete pro-inflammatory cytokines, such as IL-1, IL-6, and TNF-α, and mediate a host defense against debris and foreign materials, while M2 macrophages have been reported to contribute to tissue repair by producing anti-inflammatory cytokines such as VEGF, TGF-β and IL-10 [29]. Previous studies demonstrated a unique polarization status of macrophages by comparing regenerative activities with scar-forming activities [25]. To understand how macrophages participate in skin regeneration, we sought to identify the phenotypes of macrophages during tissue expansion. We found M1 macrophages were dominant following silicone expander implantation, suggesting early inflammation occurred after the surgery. In contrast, the number of M2 macrophages maintained in the high level during tissue expansion and skin regeneration, suggesting mechanical tension provide microenvironment more suitable for M2 macrophage polarization.

Several studies have shown that growth factors were involved in cell proliferation and vascularization during tissue expansion [7, 14]. Moreover, M2 macrophages are reported to play a role in angiogenesis and collagen synthesis, which associated with the cellular expressions of VEGF, bFGF, and TGF-β [30, 31]. Therefore, the transcriptional differences of growth factors between the control expanded skin and the macrophages depleted skin were examined in our study by qPCR. Higher expressions of VEGF, EGF, bFGF, and TGF-β were observed in the control expanded skin than that of the macrophages depleted skin at day 14, suggesting that macrophages might inhibit skin generation by changing the profiles of growth factors. On day 35, however, only bFGF showed a decrease in the expression level in the macrophages depleted skin. This is probably because the number of macrophages increased at day 35 compared with that at day 14 in the macrophages depleted skin (Fig. 2d).

The chronic inflammation is thought to associate with compromised healing and fibrotic disease in mammals [32]. Although many studies demonstrate that inflammation controls regeneration in Xenopus hind limb and an inflammation is necessary to induce zebrafish neurogenesis following injury, the link between “advanced” immune system of mammals and regeneration still ill-defined [33, 34]. Using a rat model of tissue expansion, our study provides evidence that macrophages play a role in skin regeneration. By further comparison on the immunological characteristics between the expanded and non-expanded skin, it may be possible to understand the underlying mechanisms involved in the skin regeneration and to develop therapies to enhance skin regeneration.

## Conclusions

Overall, our findings suggest that macrophages are necessary for skin regeneration during tissue expansion. Modulating inflammation may provide a key therapeutic strategy to promote skin growth under mechanical strain.

## Additional file

**Additional file 1.** Complications in macrophage-depleted side of the expanded skin. (A) Expander exposure was observed in macrophage-depleted side (1/8) and all control sides were normal (0/8) at day 35. (B, C) The front and the reverse side of the expanded flap. PBS-lipo, PBS liposomes; Clo-lipo, clodronate liposomes.

## Abbreviations

EGF: epidermal growth factor
bFGF: basic fibroblast growth factor
VEGF: vascular endothelial growth factor
TGF-β: transforming growth factor, beta
H&E: hematoxylin and eosin
PCNA: proliferating cell nuclear antigen
TcPO_2_: transcutaneous oxygen pressure
qPCR: quantitative real-time polymerase chain reaction
TNF-α: tumor necrosis factor-α
IL: interleukin
