# Supplementary figures and images for: Macrophages are necessary for skin regeneration during tissue expansion
Source: J Transl Med. 2019 Jan 21;17:36. doi: 10.1186/s12967-019-1780-z (PMC6341552; doi:10.1186/s12967-019-1780-z)

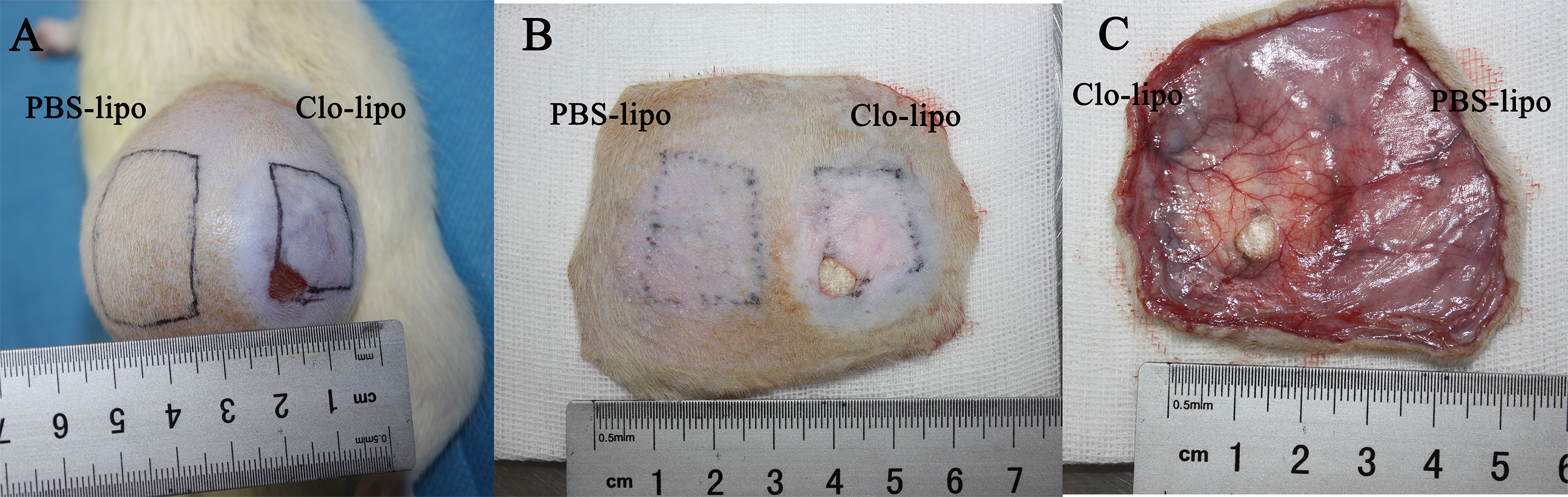

Supplement: Supplementary file 1 — Additional file 1. Complications in macrophage-depleted side of the expanded skin. (A) Expander exposure was observed in macrophage-depleted side (1/8) and all control sides were normal (0/8) at day 35. (B, C) The front and the reverse side of the expanded flap. PBS-lipo, PBS liposomes; Clo-lipo, clodronate liposomes. [file 12967_2019_1780_MOESM1_ESM.tif]
